# Yield and quality of alfalfa (*Medicago sativa* L.) in response to fertilizer application in China: A meta-analysis

**DOI:** 10.3389/fpls.2022.1051725

**Published:** 2022-11-23

**Authors:** Weifan Wan, Yuejin Li, Haigang Li

**Affiliations:** Inner Mongolia Key Laboratory of Soil Quality and Nutrient Resources, Key Laboratory of Agricultural Ecological Security and Green Development at Universities of Inner Mongolia Autonomous Region, Inner Mongolia Agricultural University, Hohhot, China

**Keywords:** fertilizer application, alfalfa, yield, quality, crude protein, acid detergent fibre, neutral detergent fibre, meta-analysis

## Abstract

**Introduction:**

In China, alfalfa (*Medicago sativa* L.) often grows in marginal land with poor soil fertility and suboptimal climate conditions. Alfalfa production cannot meet demands both in yield and quality. It is necessary to apply fertilizers to achieve high yields and produce high-quality alfalfa in China. However, there is no understanding on the impact of fertilizer application on alfalfa production and the possible optimal application rates across China.

**Methods:**

We conducted a meta-analysis to explore the contribution of fertilizer application to the yield and quality of alfalfa based on a dataset from 86 studies published between 2004 and 2022.

**Results and Discussion:**

The results showed that fertilizer application not only increased alfalfa yield by 19.2% but also improved alfalfa quality by increasing crude protein (CP) by 7.7% and decreasing acid detergent fibre by 2.9% and neutral detergent fibre by 1.8% overall compared to the non-fertilizer control levels. The combined nitrogen (N), phosphorus (P) and potassium (K) and combined NP fertilizer applications achieved the greatest yield and CP concentration increases of 27.0% and 13.5%, respectively. Considering both yield and quality, the optimal rate of fertilizer application ranged from 30 to 60 kg ha^-1^ for N, 120 to 150 kg ha^-1^ for P and less than 120 kg ha^-1^ for K. Meta-analysis further showed that the effect of fertilizer application on yield was greater in low soil organic matter (SOM) soils than in high SOM soils. In conclusion, fertilizer application is an effective strategy to improve the yield and quality of alfalfa in China, especially that grown in low SOM soils. This study is helpful for optimizing fertilization schedules of alfalfa in China.

## Introduction

Alfalfa (*Medicago sativa* L.), as a high-quality forage legume, has been cultivated on more than 4×10^5^ ha in China since 2016 (Guo et al., 2018). In recent years, alfalfa production in China cannot meet demands of the increasing animal husbandry both in yield and quality (Raiesi, 2007; Su, 2007; Zhang et al., 2009).

China’s alfalfa import increases gradually, reaching 49% of the total demand in 2015 (National Livestock stations, 2016). The yield and quality of alfalfa in China are much lower than those in developed countries, such as the USA (Wang, 2017; Wang and Zou, 2020). China Grass Industry Statistics reports that China produced 32.17 million tons of alfalfa hay, of which only 1.8 million tons of alfalfa were considered high quality in 2015. In contrast, the production of high-quality alfalfa hay reached 52.60 million tons in the USA (Wang and Zou, 2020). One of the important reasons for this discrepancy is that alfalfa must be sown in marginal land, which usually has poor soil fertility and suboptimal climate conditions (Jia et al., 2006; Fan et al., 2016; Li and He, 2017; Sun et al., 2022). Better land is used to plant grain crops to ensure food security in China (Xu et al., 2017).

Thus, fertilizer application is a direct and effective management strategy to improve the yield and quality of alfalfa, especially in low-fertility soils (Jia et al., 2006; Barker and Culman, 2020; Wang et al., 2021). However, only 57% of farmers apply fertilizers for alfalfa production in the main alfalfa production areas in China according to a survey conducted in 2013 (Xie et al., 2016). Nitrogen (N) fertilizer is applied more widely than phosphorus (P) and potassium (K) fertilizers. Many studies have been conducted on the contributions of fertilizer application to alfalfa yield and quality (Undersander et al., 2011; Macolino et al., 2013; Fang et al., 2021). However, due to differences in cultivars, soils and climate conditions, the results show large variations or even opposite results.

N fertilizer application increased the content of crude protein (CP) of alfalfa when the application rate was greater than 100 kg ha^-1^ (Oliveira et al., 2004; Zhang et al., 2020), while excessive N fertilizer may result in a higher nonprotein N content and further decreases alfalfa CP, as well as reduce N fixation capacity (Hojjati et al., 1978; Atanasova, 2008; Bahulikar et al., 2020). In addition farmers often decide the application rate of N fertilizer according to maize (*Zea mays* L.) or wheat (*Triticum aestivum* L.) without considering N fixation of alfalfa in China. It leads to 15-22% and 11-48% applied N losses by leaching and ammonia volatilization from soil, respectively (Cai et al., 2002; Zhou & Butterbach-Bahl, 2013; Li & He, 2017).

Alfalfa yield increases linearly with P application without other management measures (Gu et al., 2018). Besides CP, acid detergent fibre (ADF) and neutral detergent fibre (NDF) of alfalfa also reflect its nutritional quality (Koenig et al., 1999; Robinson, 1999). A study shows a low P fertilizer application rate (25 kg ha^-1^) can increase the content of CP significantly (Lissbrant et al., 2009). In contrast, the much higher application rates (more than 100 kg ha^-1^) are needed to increase the content of CP and reduce the content of NDF and ADF of alfalfa in many other studies (Macolino et al., 2013; Yu et al., 2018).

Similarly, the effects of K fertilizer on alfalfa yield and quality were not consistent either. Lissbrant et al. (2009) and Jungers et al. (2019) found that K fertilizer application increase the content of ADF and NDF and further decrease alfalfa quality due to a large stem increase, while Macolino et al. (2013) found that K fertilizer application has no impact on ADF and NDF.

These contradictory findings on effect of fertilizer application on yield and quality of alfalfa led to a confusion to guide farmers’ alfalfa production. In addition, some studies showed that soil organic matter (SOM) can influence effect of fertilizer application (Su, 2007; Fan et al., 2013). To date, no comprehensive studies have evaluated the contribution of fertilizer application to the yield and quality of alfalfa across China. To clarify the confusion, we conducted a pairwise meta-analysis approach to (1) assess the contribution of fertilizer application to alfalfa yield and quality, (2) determine the optimal ranges of fertilizer application.

## Materials and methods

### Literature survey and data extraction

A systematic literature search was conducted in the Web of Science and China National Knowledge Infrastructure (CNKI) with various combinations of the search terms in both English and Chinese, including “alfalfa fertilization”, “alfalfa N fertilizer”, “alfalfa P fertilizer”, “alfalfa K fertilizer”, “yield” and “quality” in article title, abstract, or keywords during 2004-2022. Three criteria were used to exclude studies not relevant to this study: (1) alfalfa must have been solely cultivated in the field; (2) the studies had to report the yield or quality data of both the control treatment and fertilization treatments; and (3) studies must have been conducted in mainland China. The final dataset included 86 studies with 1731 paired observations for alfalfa yield, 1065 paired observations for alfalfa CP and 707 paired observations for alfalfa ADF and NDF. It included 51 peer-reviewed publications, 35 MSc and PhD theses. The means and standard deviations (SD) of yield and quality as well as sample sizes (n) were obtained from both fertilized treatments and unfertilized controls in each study. The SD was obtained as the product of the standard error (SE) and square root of sample size if only the SE was provided.

Missing standard deviations were calculated using the tenth of the mean of the dataset (Luo et al., 2006). In general, literature data are presented in two forms: tabular and bar charts. Tabular data was extracted directly; for data rendered as a histogram, Engauge Digitizer 10.8 (Mark Mitchell, Baurzhan Muftakhidinov and Tobias Winchen et al., “Engauge Digitizer Software.” http://markummitchell.github.io/engauge-digitizer) was used for extraction.

### Statistics

A mixed-effects model adopting a weighted resampling approach was used to assess the alfalfa yield and quality in response to N, P, and K fertilizer singly or in combined applications. Specifically, the N/P/K fertilization treatment results were divided by those for the pairwise no fertilizer controls as response ratios (R), which were further natural log-transformed to effect sizes by Equation (1).


(1) $$Ln(R)=Ln({\text{X}}_{\text{t}})-Ln({\text{X}}_{\text{C}})$$

Where lnR is the effect size, and X_t_ and X_c_ are the means of the treatment and control, respectively (Hedges et al., 1999). The lnR was further weighted by the pooled variance (v) (Equation (2)):


(2) $$v=\frac{SD_{t}^{2}}{n_{t}X_{t}^{2}}+\frac{SD_{c}^{2}}{n_{c}X_{c}^{2}}$$

Where SDt and SDc are the SDs of the treatment and control. n_t_ and n_c_ are amount of replicate for the treatment and control. In addition, we calculated the grand E++ and a bias-corrected 95% confidence interval (CI) of lnR using MetaWin 2.0 (Rosenberg et al., 1999). And we tested stability of the results through sensitivity analysis (**Table S1**). To facilitate explanation, the percent change was computed as follows (Equation (3)):


(3) Change rete=(exp (Ln (R) − 1)×100%

Because the objective of this meta-analysis was to determine how fertilization affects the yields and quality of alfalfa, N, P, and K inputs were grouped into different categories in a gradient to determine whether different categories were significantly different in E++ and CI of lnR. Each different category had control level. Five grouped moderators were determined: 1) different fertilizer types; 2) N fertilizer application rates; 3) P fertilizer application rates (as P_2_O_5_); 4) K fertilizer application rates (as K_2_O); and 5) different SOM contents. The pair data of fertilizer application was from treatments with and without certain nutrient but the same for other nutrient. For example, the pair data of N fertilizer application included N/no fertilizer, NP/P, NK/K, and NPK/PK, and the same was applied for P and K inputs. Overall and its error bars in figures were the mean effect value and 95% CIs of yield or quality change. If there was no overlap of bias-corrected 95% CIs with zero or each other, the E++ among different categories was considered to be significantly different at α = 0.05 (Gurevitch et al., 2018). A Funnel plot was plotted to test the effect of publication bias. X-axis shows the effect size ln (R) and Y-axis presents the inverse standard error of the effect size as an index of precision (Egger et al., 1997). There was light bias risk in the article, but studies were nearly symmetrically distributed around the mean effect size and data was highly precision, presenting a random sampling error (**Figure S7**) (Liu, 2011).

## Results

### Alfalfa yield in response to fertilizer application

**Figure 1:**
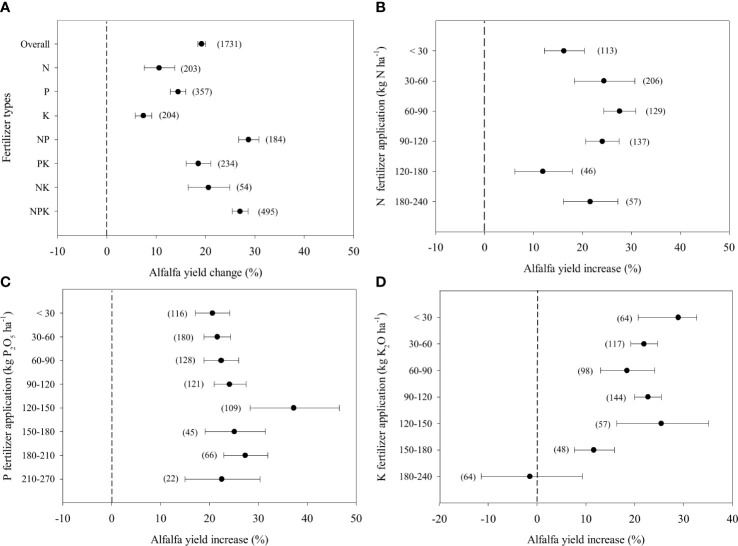
Responses alfalfa yield to different fertilizer combined application **(A)** and different rates of N **(B)**, P (as P_2_O_5_) **(C)**, K (as K_2_O) **(D)** fertilizer, expressed as the mean effect size with bias-corrected 95% confidence intervals. The number of observations is indicated in parentheses.

Overall, fertilizer application increased alfalfa yield by 19.2% compared to the control on average (**Figure 1A**). Single N, P and K fertilizer applications increased alfalfa yield by 10.6%, 14.4% and 7.4%, respectively. Combined NPK fertilizer application had the greatest effect, with a yield increase of 27.0%. With a low rate of N fertilizer application (<30 kg N ha^-1^), alfalfa yield increased by 16.2% (**Figure 1B**). The increase in alfalfa yield was the highest, reaching 27.6% at N fertilizer application rates ranged from 60 to90 kg ha^-1^. Above this application rate, the increment of alfalfa yield started to decrease. A higher P application rate than N was needed to achieve the highest increase in alfalfa yield (37.3%), which ranged from 120 to 150 kg ha^-1^ (**Figure 1C**). K fertilizer application increased alfalfa yield by approximately 25.4% when the application rate was less than 150 kg ha^-1^, above which the effect of K application declined greatly and even became negative effect when K rate ranged from 180-240 kg ha^-1^ (**Figure 1D**).

### Alfalfa CP in response to fertilizer application

**Figure 2:**
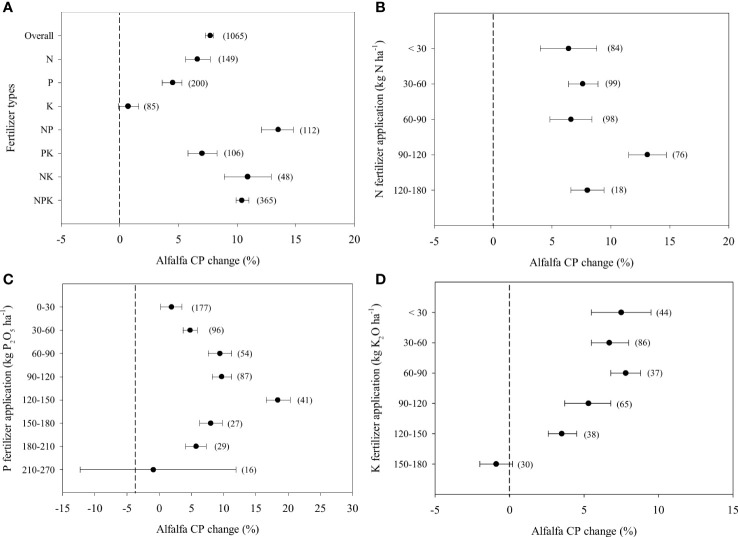
Responses alfalfa Crude Protein-CP to different fertilizer combined application **(A)** and different rates of N **(B)**, P (as P_2_O_5_) **(C)**, K (as K_2_O) **(D)** fertilizer, expressed as the mean effect size with bias-corrected 95% confidence intervals. The number of observations is indicated in parentheses.

Overall, fertilizer application increased alfalfa CP by 7.7% (**Figure 2A**). Combined NP fertilizer application achieved the greatest increase in CP concentration by 13.5%. In contrast, a single K fertilizer application had no effect on alfalfa CP. A single N fertilizer application led to a 6.6% increase in alfalfa CP, which was higher than that achieved with the single P and K fertilizer applications. The increase in alfalfa CP (13.1%) was greatest when the N application rate ranged from 90-120 kg ha^-1^ (**Figure 2B**). With increasing application rates, the effect of P fertilizers on the increase in CP content showed a unimodal pattern. The peak was 18.4% at application rates ranged from 120 to 150 kg ha^-1^, which was almost 3 to 4 times as high as that under the other rates (**Figure 2C**). Moreover, the effect of K fertilizer application on CP concentration showed a similar pattern yield. The positive effect declined to nil when excessive K fertilizer was applied (>150 kg ha^-1^) (**Figure 2D**).

### Alfalfa ADF in response to fertilizer application

**Figure 3:**
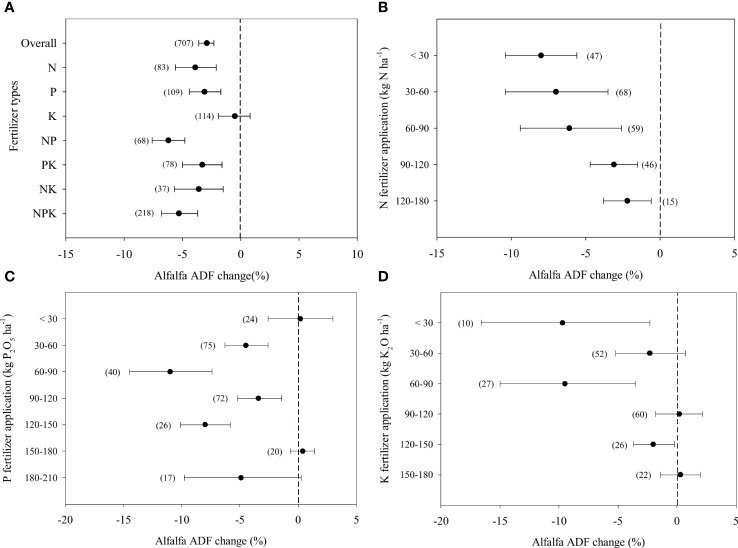
Responses alfalfa Acid Detergent Fibre-ADF to different fertilizer combined application **(A)** and different rates of N **(B)**, P (as P_2_O_5_) **(C)**, K (as K_2_O) **(D)** fertilizer, expressed as the mean effect size with bias-corrected 95% confidence intervals. The number of observations is indicated in parentheses.

Fertilizer application decreased alfalfa ADF by 2.9% on average (**Figure 3A**). The effects were similar to those of single N and P fertilizer application, but single K fertilizer application had no effect on ADF. The greatest decrease in ADF was 5.3% when NPK fertilizers were applied in combination. The increasing application rate reduced the negative effect on the ADF concentration of alfalfa brought by N fertilizer application (**Figure 3B**). The benefits became nil when the application rate reached 90 kg ha^-1^. When P fertilizer application rates ranged from 30 to 150 kg ha^-1^, the ADF concentration of alfalfa decreased by 4.5%-11% (**Figure 3C**). The decrease of alfalfa ADF was greater by 9.7 and 9.5% when K application rate ranged from<30 and 60-90 kg ha^-1^ (**Figure 3D**).

### Alfalfa NDF in response to fertilizer application

**Figure 4:**
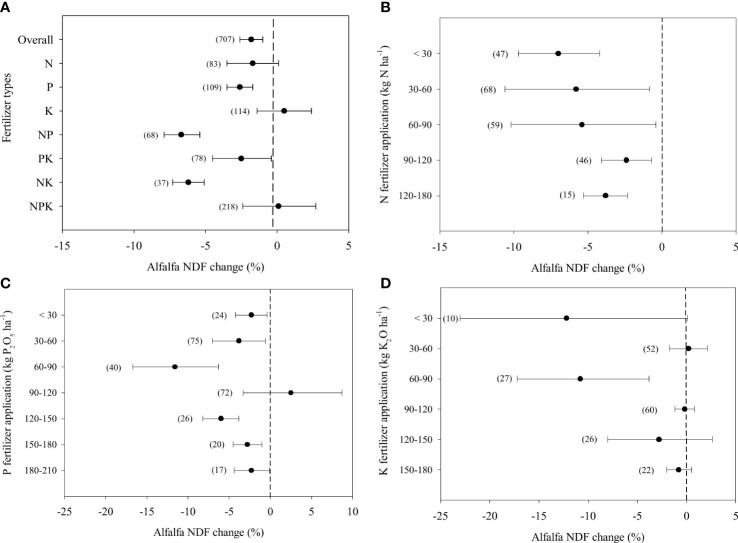
Responses alfalfa Neutral Detergent Fibre-NDF to different fertilizer combined application **(A)** and different level of N **(B)**, P (as P_2_O_5_) **(C)**, K (as K_2_O) **(D)** fertilizer, expressed as the mean effect size with bias-corrected 95% confidence intervals. The number of observations is indicated in parentheses.

Overall, fertilizer application decreased the NDF concentration of alfalfa by 1.8% (**Figure 4A**). Single N and K fertilizers and combined NPK application did not change the NDF concentration. While single P and combined NP, PK, NK fertilizer application decreased the NDF concentration of alfalfa by 2.6%, 6.7%, 2.5% and 6.2%, respectively. The low N fertilizer application rate (< 30 kg ha^-1^) led to the greatest decrease in alfalfa NDF concentration, which was similar to the trend of ADF response (**Figure 4B**). Additionally, similar to ADF, the NDF concentration decreased by 11.6% at the middle P supply rate (60-90 kg ha^-1^), which was the greatest response among the application rates (**Figure 4C**). When the rates were higher than 180 kg ha^-1^, the NDF concentration no longer responded to P fertilizer application. The K fertilizer application had less effect on alfalfa NDF concentration than N and P. Alfalfa NDF showed a significant decrease by 10.8% only at K fertilizer application rates ranged from 60 to 90 kg ha^−1^. At the other rates, K fertilizer application did not change the alfalfa NDF concentration (**Figure 4D**).

### Effect of fertilizer application on the yield and quality of alfalfa in response to SOM

**Figure 5:**
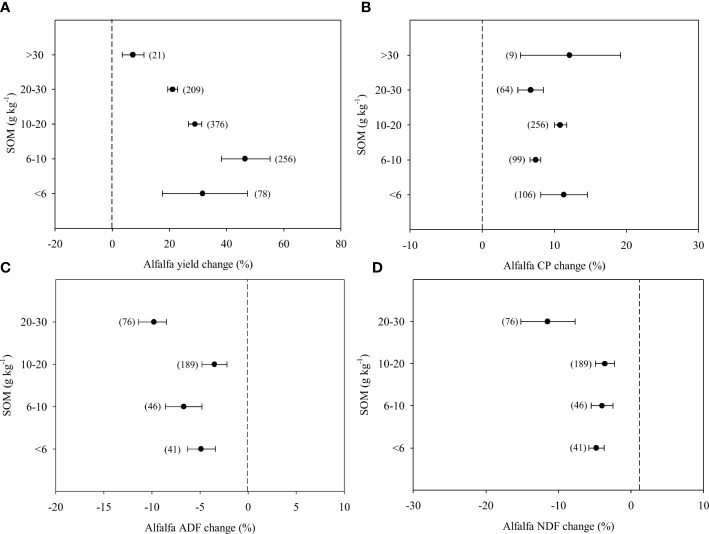
Response of yield **(A)**, Crude Protein-CP **(B)**, Acid Detergent Fibre-ADF **(C)** and Neutral Detergent Fibre-NDF **(D)** of alfalfa to different SOM: Soil organic matter (g kg^-1^), expressed as the mean effect size with bias-corrected 95% confidence intervals. The number of observations is indicated in parentheses.

The degree of the effect of fertilizer application on the yield and quality of alfalfa depended on SOM (**Figure 5**).The positive effect of fertilizer application on alfalfa yield declined with increasing SOM content (**Figure 5A**). When the SOM was less than 10 g kg^-1^, the positive effect of fertilizer application was 46.4%, while it decreased to 7.2% when SOM was greater than 30 g kg^-1^ SOM. The CP increase for the additional fertilizer application increased to 6.0-12.0% and was no difference among different SOM contents (**Figure 5B**). In contrast, high (> 20 g kg^-1^) SOM contents enhanced the decrease in ADF and NDF caused by fertilizer application (**Figures 5C, D**). When SOM was higher than 20 g kg^-1^, the decrease in ADF was 9.8%, which was almost higher than that below at >20 g kg^-1^ SOM (**Figure 5C**). The NDF concentration decreased by 11.5% with fertilizer application when the SOM was higher than 20 g kg^-1^ and was almost 2 to 3 times as high as that at the other SOM levels (**Figure 5D**).

## Discussion

### Alfalfa yield in response to fertilizer application

Alfalfa is mow 2 to 4 times each year at the early flowering stage and alfalfa removes large amounts of N, P and K per hectare from the soil each year (Kelling, 2000). All fertilizer applications significantly increased alfalfa yields in China (**Figure 1A**). This result suggested that fertilizer application is necessary for alfalfa production in China. Our research also found that combined NPK fertilizer applications resulted in the largest improvement in yield, especially in castanozems, gray desert soils and sierozem by 20-60% (**Figure S3**). These results confirm the previous study of Cai et al. (2021), which showed that there is the greatest yield increase when NPK is combined applied compared with the other single nutrient application. In addition, photosynthesis, root activity and cold resistance of alfalfa are promoted by NPK fertilizer combined application (Qamar et al., 2005; Tautges et al., 2018; Jungers et al., 2019), thereby further significantly increasing the alfalfa yield (Li, 2007; Xiao et al., 2016; Zhang et al., 2020). The proportions of applied N, P and K are expected to influence nutrient uptake and production of alfalfa. However, we cannot estimate this influence because data are not sufficient in different groups in this study. Approximately 88.8% of alfalfa production is located in North and Northwest China (Lin et al., 2017), where soil K bioavailability is often high, ranging from 76.8 to 118.0 mg L^-1^ (He et al., 2015). And our result also confirmed it (**Figure S2A**). It was the reason why single K application had the weak effect on yield (**Figure 1A**, Li and He, 2017).

Although biological N fixation supplies almost 60-85% of the N demand of alfalfa (Hannaway and Shuler, 1993; Peoples et al., 2009), starter fertilizer is still necessary to help nodule formation during the establishment phase (Koenig et al., 1999; Oliveira et al., 2004; Ghiocel et al., 2013). Moreover, to close the gap between N demand and N fixation, some N fertilizer must be applied to soil, and the amount can estimate based on N use efficiency (Mondal et al., 2020). It was confirmed by our results that N application at a rate of 30-120 kg ha^-1^ can significantly increase alfalfa yield compared with less than 30 kg ha^-1^ in China, but a further positive effect was not observed when the rate exceeded 120 kg ha^-1^ (**Figure 1B**). It indicated that Chinese farmers should put less than 120 kg ha^-1^ N fertilizer application to avoid benefit lost during alfalfa production. Moreover, the high N input also enhances losses through leaching, volatilization and denitrification (Barker and Culman, 2020; Zeng et al., 2021).

In most alfalfa-planted soils, soil Olsen-P is lower than 10 mg kg ^-1^ in China, which is defined as the critical level for alfalfa growth (He et al., 2014). Thus, P application increased alfalfa yield at all rates in China (**Figure 1C**). The alfalfa branches, chlorophyll content of leaves and photosynthetic rate increase with P fertilizer application (Macolino et al., 2013). However, a unimodal curve between alfalfa yield and P application rate is shown in a previous study (Yin et al., 2019). Our results confirmed that alfalfa reached the highest yield at a rate of 120-150 kg ha^-1^ (**Figure 1C**). Excessive P application resulted in a reduced stimulatory effect on yield, which was due to a certain threshold of P absorption by alfalfa plants (Yu et al., 2018). Like N, excessive P application does not result in a higher yield and cost P fertilizer waste. The remaining P in soil was fixed quickly by soil minerals and because P is unavailable for alfalfa in most soils (Urrutia et al., 2013). Moreover, soil Olsen P level also influences yield response to fertilizer application. It reached to the greatest when soil Olsen P ranged from 3 mg kg^-1^ to 20 mg kg^-1^ (**Figure S1A**).

Our result showed that excessive K application reduced the positive effect on yield (**Figure 1D**). As the discussion above, high K bioavailability in soils of North and Northwest China limited the positive effect of K fertilizer application on alfalfa yield and quality (**Figures 1A**, **S2A**, He et al., 2015; Lin et al., 2017). Moreover, Lloveras et al. (2012) reported that high K fertilizer application may disturb the uptake of other macronutrients, such as N.

### The effect of fertilizer application on alfalfa quality

Fertilizer applications significantly improved alfalfa CP in China regardless of soil Olsen P and soil K supply levels (**Figures 2A**, **S1B**, **S2B**). N fertilizer was more beneficial to the improvement of protein than single P and single K (**Figure 2A**). Single P application had a negative effect on alfalfa CP in cinnamon soils and cultivated loessal soils (**Figures S4A, B**). This is due to N application more directly stimulates the key enzymes of N metabolism and further promotes synthesis of proteins, such as glutamine synthase (GS) and glutamate synthase (GOGAT) (Geisseler et al., 2010; Shah et al., 2017). Phosphorus facilitates N accumulation and CP synthesis of alfalfa by increasing N fixation efficiency (Macolino et al., 2013). But P deficiency stress significantly decreases the shoot CP concentration in soybean (Qiao et al., 2007), which was consistent with our results. Thus, all N or P fertilizer applications increased the shoot CP concentration. Single K fertilizer application had little impact on the shoot CP concentration of alfalfa (**Figure 2A**), and even reduced alfalfa CP in castanozems, irrigation desert soils and gray desert soils (**Figures S4A, B**). The reduction in the CP response to K fertilization attributes to a reduction in the leaf/shoot ratio and an increase in shoot biomass (Berg et al., 2005).

The shoot CP concentration of alfalfa was more sensitive to N fertilizer application than yield. Even a low N fertilizer application rate (<30 kg ha^-1^) led to a similar increase in shoot CP concentration at a rate of 30-90 kg ha^-1^ (**Figure 2B**). As the improving metabolism is always a higher priority than biomass accumulation for N-deficient plants (Schluter et al., 2012). In the present research, N input at 90-120 kg ha^-1^ could significantly improve alfalfa CP and did not have a further positive effect when it exceeded 120 kg ha^-1^ (**Figure 2B**), which might be because the synthesis of amino acids and protein N increase with the increase in the N application rate. However, excessive application of N fertilizers result in a significant reduction in protein N compare to increase free amino acids and nonprotein N (Atanasova, 2008).

The CP content of alfalfa showed a unimodal trend with increasing P application, and the optimal application rate was 120-150 kg ha^-1^ (**Figure 2C**). This is due to an increase in N utilization and metabolism rates with increasing P inputs (Qiao et al., 2007; Rashid and Iqbal, 2012). P deficiency reduce N fixation, photosynthesis and leaf area (Sanginga, 2003; Liu et al., 2020). However, excessive phosphate fertilizer causes the increase in respiration, which depletes store sugar and energy. Sequentially, it causes alfalfa CP to tend to decline (Patrick et al., 2013). The application of K does not always promote CP synthesis (**Figure 2D**). High K application increase lignification and further lead to a decline in the CP content (Barker and Culman, 2020). Optimal K application is necessary to avoid the decline of alfalfa quality caused by stem/leaf ratio increase. Soil K supply should be also considered if farmers took account of the economic benefit according to our result (**Figure S2B**).

The N and P fertilizer application reduces cell wall concentrations and lignin concentrations by promoting plant metabolism (Parsons et al., 2009). This is why alfalfa ADF and NDF concentrations decreased significantly in treatments containing N and P fertilizers application although a few data were exceptive (**Figure 3A**, **S1C, S1D, S2C, S2D**). K did not have the same function as N and P. Thus, single K fertilizer application had no effect on alfalfa ADF and NDF concentrations (**Figure 3A**).

However, excessive N fertilizer application increases the lignification of alfalfa (Unkovich, 2012), which is supposed to offset the positive effect of N fertilizer application on ADF and NDF concentrations. Our results confirmed that ADF and NDF concentrations did not change when the N application rate was greater than 90 kg ha^-1^ (**Figures 3B**, **4B**). Although the effects of P fertilizer application on ADF and NDF concentrations varied greatly, there is no doubt that an appropriate moderate P application rate significantly reduces ADF and NDF concentrations (**Figures 3C**, **4C**). This was due to an increase in the number of branches and a decrease in the ratio of stems: leaves (Lissbrant et al., 2009), which does not change significantly when alfalfa is supplied with low or extremely high P fertilizer rates (Zhang et al., 2020). Thus, the amount of P fertilizer applied should be 30-150 kg to change the NDF and ADF. In most K fertilization rates, K had no effect on ADF and NDF (**Figures 3D**, **4D**, **S5, 6**). Due to the high soil K concentrations in soil, the effect of K fertilizer application on forage nutritive value might not improve (Jungers et al., 2019). Moreover, K improve the cell wall concentration and increased the stem fibre digestibility or cell wall composition and concentration (Lamb et al., 2012).

### The contribution of SOM to the improvement of alfalfa yield and quality

Our results showed that the SOM content was mainly responsible for the different effects of fertilizer application on alfalfa yield and quality (**Figure 5**), which was consistent with the results on cereal crops by Fan et al. (2013). SOM is more important for fertilizer application effects in low-fertility soils than that in high-fertility soils. Mineralization of SOM can release nutrients into soils to replenish nutrient removal by plant adsorption, which is the reason why the fertilizer application effect became weaker in high SOM soils (Li et al., 2008; Oldfield et al., 2019; Du et al., 2020). However, the effect of SOM on wheat yield disappears when the SOM content is more than 15 g kg^-1^ (Fan et al., 2013). In this study, high SOM (> 20 g kg^-1^) still significantly enhanced the effects of fertilizer application on ADF and NDF (**Figures 5C, D**). This result was possibly due to the greater number of leaves and low lignification when alfalfa grown in these soils (Jung and Engels, 2002; Jia et al., 2006; Vasileva and Kostov, 2015).

## Conclusions

Fertilizer application not only increased alfalfa yield by 19.2% but also improved alfalfa quality by increasing CP of 7.7% and decreasing ADF of 2.9% and NDF of 1.8% compared to the non-fertilizer control levels without any fertilizer application, especially in low SOM soils. The optimal N, P and K fertilizer application rates for alfalfa production in China were 30-90 kg ha^−1^, 30-150 kg ha^−1^ and 0-120 kg ha^−1^, respectively, according to a comprehensive data analysis. The combined NPK and NP fertilizer application had the best effect on improving alfalfa yield and quality. The results provided a guide to optimize nutrient management on alfalfa production in China by clarifying the current confusions of fertilizer application.

## Data availability statement

The original contributions presented in the study are included in the article/**Supplementary Material**. Further inquiries can be directed to the corresponding authors.

## Author contributions

WW performed the data collection, analyses and wrote the manuscript; HL and YL helped perform the analysis with constructive discussions. All authors contributed to the article and approved the submitted version.

## Funding

This study was supported by grant from the Double First-Class Financial Capital in China (Grant no.: NDYB2018-4), the Scientific Research Start-up Fund of the Autonomous Region Human Resources and Social Security Department in 2018 (for HL), Project of Grassland Talent (for HL).

## Conflict of interest

The authors declare that the research was conducted in the absence of any commercial or financial relationships that could be construed as a potential conflict of interest.

## Publisher’s note

All claims expressed in this article are solely those of the authors and do not necessarily represent those of their affiliated organizations, or those of the publisher, the editors and the reviewers. Any product that may be evaluated in this article, or claim that may be made by its manufacturer, is not guaranteed or endorsed by the publisher.
