# Supplementary material for: Yield and quality of alfalfa (Medicago sativa L.) in response to fertilizer application in China: A meta-analysis
Source: Front Plant Sci. 2022 Nov 23;13:1051725. doi: 10.3389/fpls.2022.1051725 (PMC9728100; doi:10.3389/fpls.2022.1051725)
Supplement: Supplementary file 1 [file DataSheet_1.docx]

**Supplementary material (Figures and Tables)**

B

A

**V**

D

C

**Fig.S1** Response of yield (A), Crude Protein-CP (B), Acid Detergent Fibre-ADF (C) and Neutral Detergent Fibre-NDF (D) of alfalfa to different Soil Olsen P (g kg^-1^), expressed as the mean effect size with bias-corrected 95% confidence intervals. The number of observations is indicated in parentheses.

A

B

D

C

**Fig.S2** Response of yield (A), Crude Protein-CP (B), Acid Detergent Fibre-ADF (C) and Neutral Detergent Fibre-NDF (D) of alfalfa to different NH_4_OAc-exchangeable K (g kg^-1^), expressed as the mean effect size with bias-corrected 95% confidence intervals. The number of observations is indicated in parentheses.

B

A

**Fig.S3** Responses alfalfa yield to different fertilizer combined application (A, B), expressed as the mean effect size with bias-corrected 95% confidence intervals. The fertilizer of N, P_2_O_5_ and K_2_O are expressed as N, P and K. The number of observations is indicated in parentheses.

B

A

**Fig.S4** Responses alfalfa Crude Protein-CP to different fertilizer combined application (A, B), expressed as the mean effect size with bias-corrected 95% confidence intervals. The fertilizer of N, P_2_O_5_ and K_2_O are expressed as N, P and K. The number of observations is indicated in parentheses.

**Fig.S5** Responses alfalfa Acid Detergent Fibre-ADF to different fertilizer combined application, expressed as the mean effect size with bias-corrected 95% confidence intervals. The fertilizer of N, P_2_O_5_ and K_2_O are expressed as N, P and K. The number of observations is indicated in parentheses.

**Fig.S6** Responses alfalfa Neutral Detergent Fibre-NDF to different fertilizer combined application, expressed as the mean effect size with bias-corrected 95% confidence intervals. The fertilizer of N, P_2_O_5_ and K_2_O are expressed as N, P and K. The number of observations is indicated in parentheses.


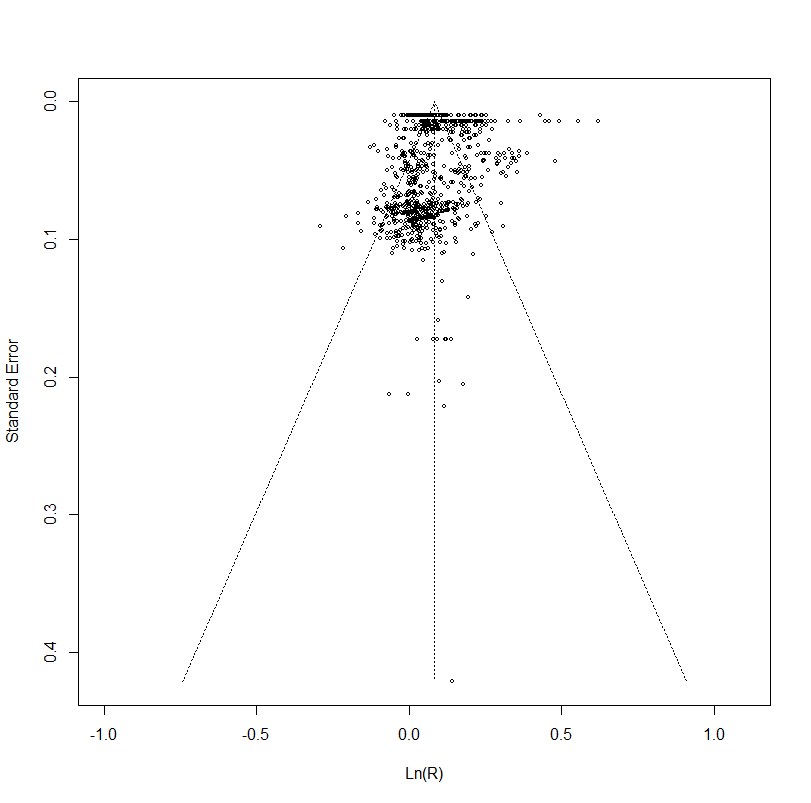

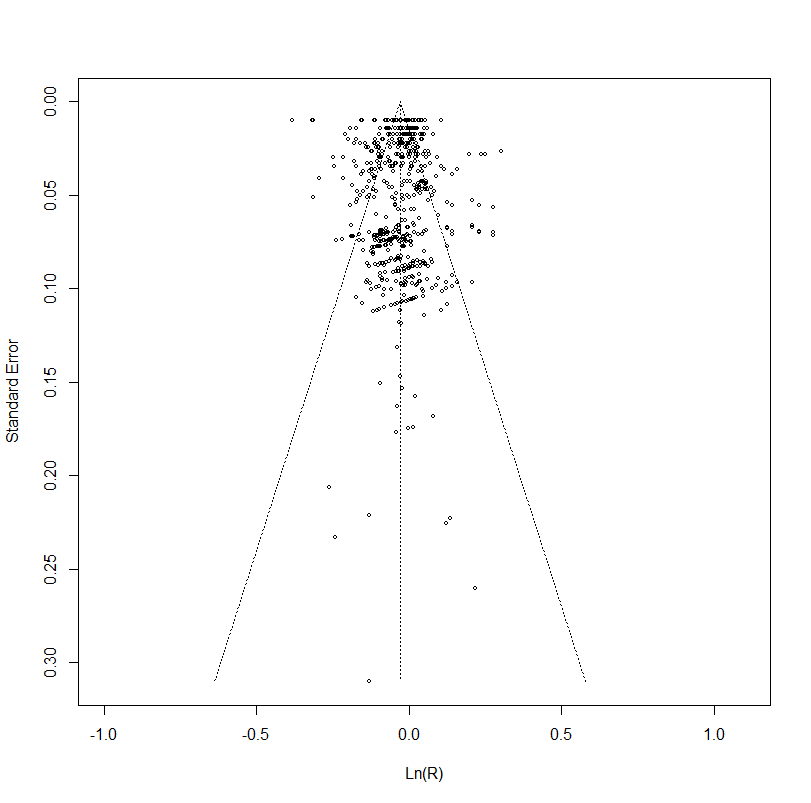

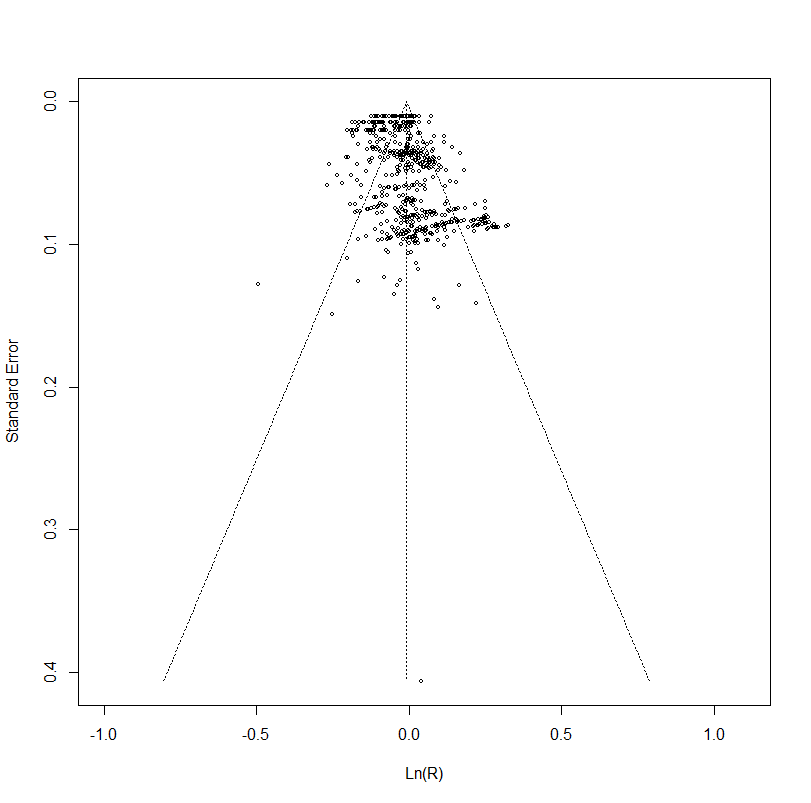

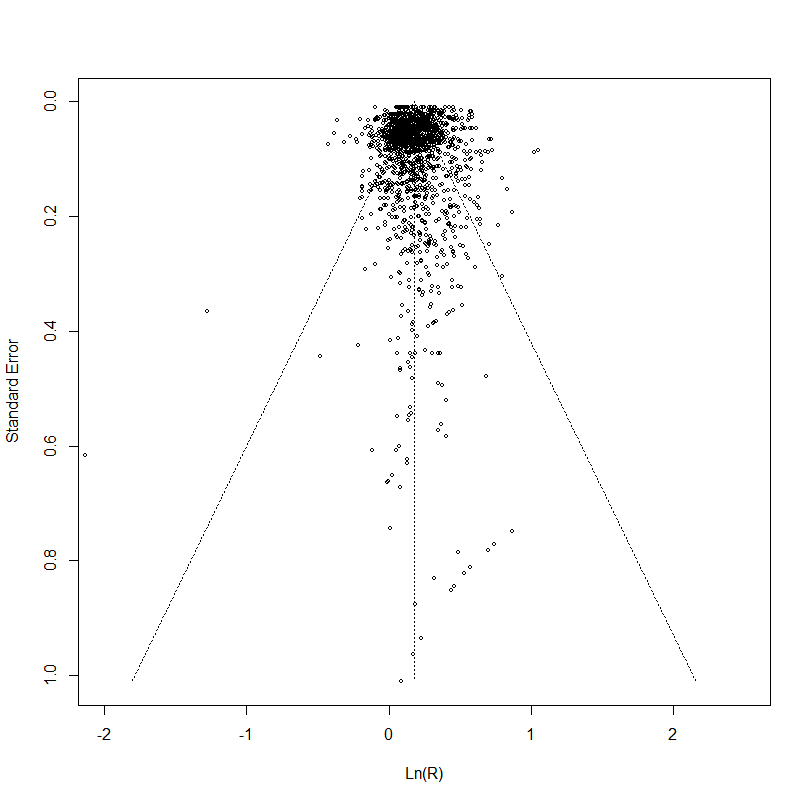


A

B

C

D

**Fig. S7** Funnel plot of standard error of yield (A), Crude Protein-CP (B), Acid Detergent Fibre-ADF (C) and Neutral Detergent Fibre-NDF (D) against response variable Ln(R). The vertical line represents the mean log response ratio Ln(R) estimated.

**Table.S1** The sensitivity analysis on alfalfa yield, CP, ADF and NDF

| Index | Fertilizer  types | Ln(R)  (%) | 95% CI  (%) | | n | Study |
| --- | --- | --- | --- | --- | --- | --- |
| Yield | N | 11.8 | 8.3 | 15.4 | 12 | Hu et al., 2019 |
|  | P | 14.4 | 9.1 | 19.9 | 12 | Li, 2017 |
|  | K | 7.3 | 5.2 | 9.3 | 24 | Zhao, 2013 |
|  | NP | 24.9 | 19.8 | 30.3 | 12 | Chen et al., 2019 |
|  | PK | 21.6 | 15.3 | 28.3 | 9 | Zhao, 2013 |
|  | NK | 15.2 | 6.9 | 24.0 | 5 | Wu, 2020 |
|  | NPK | 25.0 | 19.7 | 30.6 | 20 | Yin, 2012 |
| CP | N | 6.8 | 3.1 | 10.7 | 15 | Tian, 2010 |
|  | P | 5.5 | 3.3 | 7.7 | 6 | Sun et al., 2022 |
|  | K | 1.0 | -1.4 | 3.4 | 20 | Pan, 2017 |
|  | NP | 19.2 | 11.5 | 27.5 | 4 | Zhang, 2017 |
|  | PK | 7.0 | 2.4 | 11.9 | 5 | Wu, 2020 |
|  | NK | 12.4 | 5.4 | 19.9 | 4 | Zhang, 2005 |
|  | NPK | 12.4 | 9.3 | 15.5 | 18 | Wang et al., 2019 |
| ADF | N | -6.6 | -10.0 | -3.0 | 10 | Zhang, 2014 |
|  | P | -4.7 | -7.1 | -2.3 | 6 | Sun et al., 2022 |
|  | K | 0.1 | -2.0 | 2.2 | 9 | Liu, 2016 |
|  | NP | -9.4 | -12.1 | -4.7 | 12 | Chen et al., 2019 |
|  | PK | -4.1 | -7.1 | -0.9 | 18 | Zhao et al., 2013 |
|  | NK | -4.8 | -9.1 | -0.3 | 3 | Zhao et al., 2013 |
|  | NPK | -4.4 | -7.8 | -1.0 | 9 | Xiao, 2016 |
| NDF | N | 0.9 | -6.1 | 8.5 | 8 | Zhang et al., 2020 |
|  | P | -3.2 | -7.1 | 0.9 | 10 | Mao, 2017 |
|  | K | 0.5 | -1.8 | 2.8 | 20 | Pan, 2017 |
|  | NP | -8.3 | -11.7 | -4.8 | 8 | Zhang, 2014 |
|  | PK | -1.2 | -3.0 | -0.7 | 12 | Chen et al., 2019 |
|  | NK | 7.2 | -10.7 | -2.5 | 3 | Zhao et al., 2013 |
|  | NPK | -1.0 | -2.0 | 0.0 | 7 | Meng, 2018 |

**2 Supplementary material (Data sources)**

1. Yang Hengshan, Cao Min jian, Li Chun long, et al., Effects of superphosphate and potassium chloride fertilization on alfalfa. (In Chinese, with English abstract.) PRATACULTURAL SCIENCE, 2003, 3, 20(11): 19-22.
2. Yu Tiefeng. Study on the construction of alfalfa fertilizer model and physiological mechanism of nutrient efficiency in the aridirrigation area of northwest China. (In Chinese, with English abstract.) [Doctoral dissertation, Gansu Agricultural University], 2018.
3. Mao Xiaotao. Effects of Continuous Fertilization on Yield and Quality of AIfalfaand Soil Mineral Nutrients Under Non-irrigated Conditions. (In Chinese, with English abstract.) [Doctoral dissertation, Inner Mongolia Agricultural University], 2017.
4. Chen Yuming, Li Qian, Wang Yuxiang, Zhang Bo. Effects of N, P, K fertilizers application on yield, rhizobium and nutrient uptake and utilization of alfalfa. (In Chinese, with English abstract.) Journal of Arid Land Resources and Environment, 2019, 33(07): 174-180.
5. Sun Yanmei, Liu Xuanshuai, Zhang Qianbin, et al., Effects of phosphorus application on hay yield and phosphorus contents of alfalfa under drip irrigation. (In Chinese, with English abstract.) Acta Prataculturae Sinica, 2019, 28(3): 154-163.
6. Wen Yang. Effect of Phosphorus and Potassium Nutrition on Yield and Quality of Alfalfa (Medicago sativa L.) and Related Mechanisms. (In Chinese, with English abstract.) [Doctoral dissertation, Chinese Academy of Agricultural Sciences], 2005.
7. Liu Xiaojing, Zhang Jinxia, Ye Fang, et al., Effects of Nitrogen Application on Nitrogen Metabolism and Nitrogen Accumulation of Alfalfa (Medicago Sativa). (In Chinese, with English abstract.) Journal of Nuclear Agriculural Sciences, 2015, 29(07): 1399-1405.
8. Tian Yutao. Effect of Irrigation and Nitrogen Supply Levels on Hay Yield. Nutrition Quality and Nitrogen Utilization of Alfalfa in Shiyang .River Basin. (In Chinese, with English abstract.) [Master’s thesis, Gansu Agricultural University], 2010.
9. Zhang Fanfan. Effects of Alfalfa Production Traits and Soil Phosphorus Changes on Different Phosphorus Application Mode by Drip Irrigation in Oasis of northern Xinjiang. (In Chinese, with English abstract.) [Master’s thesis, Shihezi University], 2014.
10. Yin Hui. Effects of irrigation and nitrogen application rates on hay yield, water use efficiency of alfalfa and fertilizer nitrogen loss. (In Chinese, with English abstract.) [Master’s thesis, Gansu Agricultural University], 2012.
11. Ji Jiaofiao. Effects of Irrigation Frequency and. Fertilization Method on Alfalfa Yield and Utilization Efficiency. (In Chinese, with English abstract.) [Master’s thesis, Lanzhou University], 2018.
12. Liu Hui. Research the Preliminary Study onWater and Fertilizer Managementof Alfalfa in Salt-affected Land in Hetao Area. (In Chinese, with English abstract.) [Master’s thesis, Inner Mongolia Agricultural University], 2017.
13. Li Xingyue. Effect of Formula Fertilization on Yield and Quality of Alfalfa inthe Region of Tumote. (In Chinese, with English abstract.) [Master’s thesis, Inner Mongolia Agricultural University], 2016.
14. Wu Bo, Zhang Qingping, Zhang Jinhong, Wang Guoliang, He Feng, Sheng Yibing. Effect of Fertilization on Yield and Quality of Alfalfa in Saline-alkali Soil. (In Chinese, with English abstract.) Chinese Agricultural Science Bulletin, 2017, 33(29): 66-71.
15. Hu Wei. Zhang Yahaong, Li Peng, et al., Effects of water and nitrogen supply under drip irrigation on the production performance rate and water and nitrogen use efficiency of alfalfa. (In Chinese, with English abstract.) Acta Prataculturae Sinica, 2019, 28(2): 41-50.
16. Meng Kai, Li Xingyue, Ji Xiaoting, Han Yaqiong, et al., Effect of Nitrogen, Phosphorus and Potassium Fertilizer on Yield of Caoyuan No. 3 Alfalfa. (In Chinese, with English abstract.) Chinese Journal of Grassland, 2019, 41(03): 107-114.
17. Zhang Tiejun, Zhao Zhongxiang, Long Ruicai, et al., Study on Effects of N, P and K Fertilizers on Alfalfa Hay and Recommended Fertilizer Rate in Huang-Huai-Hai Area. (In Chinese, with English abstract.) ACTA AGRESTIA SINICA, 2019, 27(01): 243-249.
18. Mai Maitimin. Nai Yimu, Wang Yu xiang, et al., Effects of Fertilization on Yield and Quality of Xinmu No.4 Medicago Sativa L. (In Chinese, with English abstract.) Plant-eating livestock,2018(05): 43-47.
19. Wang Yang, Cui Guowen, Yin Hang, et al., Effects of different fertilization schemes on alfalfa performance and nutritional quality. (In Chinese, with English abstract.) Pratacultural Science, 2019, 36(3): 793-803.
20. Meng Yang. Effects of different fertilization treatments on yield and quality of alfalfa. (In Chinese, with English abstract.) [Master’s thesis, Hebei Agricultural University], 2018.
21. Li Chenjian, Wang Yuxiang. Zhang Bo. Proportional Fertilization: Effects on Quality and Yield of Alfalfa. (In Chinese, with English abstract.) Chinese Agricultural Science Bulletin, 2018, 34(36): 146-152.
22. Shi XiaoPeng. The effect of nitrogen and phosphorus fertilizer on Different Growing Year alfalfa aboveground biomass, soil nutrients and soil water in the semi-arid area. (In Chinese, with English abstract.) [Master’s thesis, Lanzhou University], 2018.
23. Yang HaoHong. Effects of nitrogen, phosphorus and potassium fertilizers on yield and quality of alfalfa under drip irrigation. (In Chinese, with English abstract.) [Master’s thesis, Tarim University], 2017.
24. Zhang Min. The effect of different fertilizer on the growth status and nutrient value of transgenic alfalfa expressing stress tolerance genes from Zygophyllum xanthoxylum underdrought condition. (In Chinese, with English abstract.) [Master’s thesis, Lanzhou University], 2017.
25. Ge Liaosheng, Zhang Haitao, Liu Chengjun, et al., Effects of Nitrogen, Phosphorus and Potassium Fertilization Ratio on Nutritional Quality of Alfalfa Cultivated in Alluvial Plain of the Yellow River. (In Chinese, with English abstract.) Animal Husbandry and Feed Science, 2017, 38(01): 52-54.
26. Pan Jia. Cultivation Techniques of Alfalfa on the LoessPlateau. (In Chinese, with English abstract.) [Master’s thesis, Lanzhou University], 2017.
27. Xiao Yanzi, Ge Gentu, Lv Shijie, et al., The research of "ZhongMu No. 2" alfalfa in high yield and fertilizer. (In Chinese, with English abstract.) Journal of Arid Land Resources and Environment, 2016, 30(09): 183-189.
28. Zhang Xuezhou, Lan Jiyong, Zhang Huihui, et al., Effects of Different Fertilization Ratio on Yield,Quality and Benefit of Multifoliate Alfalfa. (In Chinese, with English abstract.) Modern agricultural science and Technology, 2016(04): 270-273.
29. Xiao Xianghua. Effects of Different Ratio of Nitrogen, Phosphorus and PotassiumFertilizer on Yield, Quality and Root Nodule Bacteria Number of Alfalfa (Medicago sativa L.). (In Chinese, with English abstract.) Xinjiang Agricultural University, 2016.
30. Wang Yongning. Research on Fertilization Effect After Several Years of Alfalfain Salt-affected Land in Hetao Area. (In Chinese, with English abstract.) [Master’s thesis, Inner Mongolia Agricultural University], 2016.
31. Liu Hao. The application of the formula fertilization on alfalfa productionon Tumochuan plain. (In Chinese, with English abstract.) [Master’s thesis, Inner Mongolia Agricultural University], 2016.
32. Zhang Jing, Zhang Yuhui, Ma Li, Zhou Peng, et al., Effect of Sowing Date and Applying Fertilizer on Growth andQuality of Alfalfa in Winter Fallow Land. (In Chinese, with English abstract.) Chinese Journal of Grassland, 2015, 37(06): 35-41.
33. Xu Bo, Liu Zhuo, Wang Yingzhe, et al., Effect of Nitrogen, Phosphorus and Potassic Fertilizer on the Yield of Alfalfa (Medicago sativa L.). (In Chinese, with English abstract.) Journal of Jilin Agricultural Sciences, 2015, 40(06): 47-50+79.
34. Wang Qian, Wang Sheng Wen, Chen wei, et al., Effects of fertilization on yield, quality and economic efficiency of alfalfa in low-yield field in Mingle. (In Chinese, with English abstract.) Pratacultural Science, 2016, 33(2): 230-239.
35. Chen Ping, Shen Zhenrong, Chi Haifeng, et al., Effects of Different Fertilizing Levels on the Yield and Ecological Index of Alfalfa. (In Chinese, with English abstract.) Grass and Animal Husbandry, 2013(03): 8-11.
36. Zhang JinXia. Effect of nitrogen and phosphorus supply levels on productivity and the soil nutriment feature of alfalfa. (In Chinese, with English abstract.) [Master’s thesis, Gansu Agricultural University], 2014.
37. Liu Yarnan, Liu Xiaojing. Effect of fertilization on production performance and quality of different varieties of alfalfa. (In Chinese, with English abstract.) JOURNAL OF GANSU AGRICULTURAL UNIVERSITY, 2014, 49(01): 111-115+120.
38. Xie Yong, Sun Hongren, Zhang Xinquan, et al., Effects of N, P and K Fertilizer on Alfalfa and Recommended Fertilizer Rate in Bashang Area. (In Chinese, with English abstract.) Chinese Journal of Grassland, 2012, 34(02): 52-57.
39. Zhao Yun. Response of Alfalfa Productivity to Different Fertilization Management and Balanced Fertilization Models. (In Chinese, with English abstract.) [Doctoral dissertation, Chinese Academy of Agricultural Sciences], 2013.
40. Li Rongxia. The Influence of Different Fertilization Levels on Yields, Nutrient Absorption and Soil Fertility of Alfalfa. (In Chinese, with English abstract.) [Master’s thesis, Xinjiang Agricultural University], 2007.
41. Zhao Yun, Xie Kaiyun, Yang Xiufang, et al., Effects of nitrogen, phosphorus and potassium ratio fertilizer on the the yield and quality of Aohan alfafa. (In Chinese, with English abstract.) PRATACULTURAL SCIENCE, 2013, 30(05): 723-727.
42. Wang Congming. Effect of Fertilization on Alfalfa of High Yield and High Quality. (In Chinese, with English abstract.) [Master’s thesis, Inner Mongolia Agricultural University, 2016.
43. Zhang LiJuan. Effect of root nodule bacteria, fertilization on Alfalfa's (Medicago sativa) production performance. (In Chinese, with English abstract.) [Master’s thesis, Shanxi Agricultural University], 2005.
44. Li Yuan, Wang Zan, Gao Hongwen, et al., Effects of P and K Combined Fertilization on the Yield of Alfalfa. (In Chinese, with English abstract.) North China Journal of Agronomy, 2008, 23(S2):315-318.
45. Fan Zhidong. Analysis and Study on Water and Fertilizer Coupling Mechanism of Alfalfa in MaoWuSu Sandy Land. (In Chinese, with English abstract.) [Master’s thesis, Inner Mongolia Agricultural University], 2012.
46. Liu Yan, Liu Jianwen, Zhang Kanglin, et al., Observation on the Effect of Different Fertilization Treatments on Increasing Yield. (In Chinese, with English abstract.) Animal husbandry veterinarian of Hubei province, 2010(07): 6-8.
47. Liu Jixian, Li Wei, Effect of balanced fertilization on yield and quality of Purple flower. (In Chinese, with English abstract.) South China Agriculture, 2012, 6(05): 6-8.
48. Fan Fu, Zhang Ning, Zhang Qingguo, et al., Effects of Fertilizer Application on the Yield of Fresh Forage and Nutrient Content of Aohan Alfalfa. (In Chinese, with English abstract.) Chinese Journal of Grassland, 2007(05): 36-42.
49. Han Qingfang, Zhou Fang, Jia Jun, et al., Effect of fertilization on productivity different producing performance alfalfa varieties and soil fertility. (In Chinese, with English abstract.) Plant Nutrition and Fertilizer Science, 2009, 15(06): 1413-1418.
50. Wu Jianxin. The Effects of Fertilization on Performance of Medicago variaMartin. cv. Caoyuan No.3. (In Chinese, with English abstract.) [Master’s thesis, Inner Mongolia Agricultural University], 2007.
51. Tuo Erkun Mai Maiti, Yu Lei, Guo Jiangsong, Lin Xiang-qun. Study on the Effects of Fertilization on Yield and Quality of Two Varieties of Alfalfa. (In Chinese, with English abstract.) Xinjiang Agricultural Sciences, 2009, 46(06): 1373-1377.
52. Lin Xiangqun, Yu Lei, Lu Weihua. The effect of fertilizer on productivity of different Medicago sativa varieties in Oasis. (In Chinese, with English abstract.) PRATACULTURAL SCIENCE, 2007(09): 48-51.
53. Fan Fu, Zhang Ning, Zhang Qingguo, Tai Jicheng, Sun Dezhi, Su Yale. The Effects of N, P, K Mixed Fertilizing on Fresh Forage Yield and Nutriention of Argangjin Alfalfa. (In Chinese, with English abstract.) North China Agricultural Science Journal, 2008(04): 184-189.
54. Ma Xiaohui. The Affection of Fertilizer on Alfalfa and Analyzing of economy. (In Chinese, with English abstract.) [Master’s thesis, Xinjiang Agricultural University], 2005.
55. Zeng QingFei. Fertilization Effects on Alfalfa ProductionCharacteristics and Soil Fertility. (In Chinese, with English abstract.) [Master’s thesis, Northwest Agriculture & Forestry University], 2005.
56. Zhang Dongtie, Tang Fenglan, Zhu Ruifen, et al., Effect of Seeding Rate, Fertilizer Stage and Fertilizer Quantity on the Yield of Alfalfa on Saline-Alkali Soil. (In Chinese, with English abstract.) Heilongjiang Agricultural Sciences, 2011(12): 116-119.
57. Cao Jing, Li Xianting, Kong Xiaole R, et al., Using alfalfa (Medicago sativa) to ameliorate salt-affected soils in Yingda irrigation district in Northwest China. (In Chinese, with English abstract.) Acta Ecologica Sinica, 2012(32): 68-73. doi:10.1016/j.chnaes.2011.12.001.
58. Fan Jingwei, Du Yanlei, Wang Bingru, et al., Forage yield, soil water depletion, shoot nitrogen and phosphorus uptake and concentration, of young and old stands of alfalfa in response to nitrogen and phosphorus fertilisation in a semiarid environment. Field Crops Research, 2016(198): 247-257. doi:10.1016/j.fcr.2016.08.014.
59. Gu Yanjie, Han Chenglong, Kong Meng, et al., X.-Y. Shi, P. Zdruli and F.-M. Li. Plastic film mulch promotes high alfalfa production with phosphorus-saving and low risk of soil nitrogen loss. Field Crops Research, 2018(229): 44-54. doi:10.1016/j.fcr.2018.09.011.
60. Gu Yanjie, Han Chenglong, Fan Jingwei, et al., Alfalfa forage yield, soil water and P availability in response to plastic film mulch and P fertilization in a semiarid environment. Field Crops Research, 2018(215): 94-103. doi:10.1016/j.fcr.2017.10.010.
61. Jia Qianmin, Muhammad Kamran, Shahzad Ali, et al., Deficit irrigation and fertilization strategies to improve soil quality and alfalfa yield in arid and semi-arid areas of northern China. PeerJ, 2018(6): e4410. doi:10.7717/peerj.4410.
62. Cao Xuesong, Feng Yayang, Li Heping, et al., Effects of Subsurface Drip Irrigation on Water Consumption and Yields of Alfalfa under Different Water and Fertilizer Conditions. Journal of Sensors, 2021, 1–12. https://doi.org/10.1155/2021/6617437
63. Meng Kong, Jing Kang, Cheng-Long Han, et al., Nitrogen, Phosphorus, and Potassium Resorption Responses of Alfalfa to Increasing Soil Water and P Availability in a Semi-Arid Environment. Agronomy, 2020, 10(2): 310. https://doi.org/10.3390/agronomy10020310
64. Sun Yanliang, Wang Xuzhe, Ma Chunhui, et al., Effects of Nitrogen and Phosphorus Addition on Agronomic Characters, Photosynthetic Performance and Anatomical Structure of Alfalfa in Northern Xinjiang, China. Agronomy, 2022, 12(7): 1613. https://doi.org/10.3390/agronomy12071613
65. Zhang Qianbing, Liu Junying, Liu Xuanshuai, et al., Optimizing water and phosphorus management to improve hay yield and water‐ and phosphorus‐use efficiency in alfalfa under drip irrigation. Food Science & Nutrition, 2020, 8(5): 2406–2418.
66. Liu Qingsong, Jia Yanli, Yao Yu, et al., Effects of Balanced Fertilization on Alfalfa Growth and Soil Nutrient Changes in the Cangzhou Area. (In Chinese, with English abstract.) Chinese Journal of Grassland, 2022,44(07):70-78.
67. Li Yuan, Zhang Qingping, Wang Tao,Shen Yuying et al., Effects of nitrogen addition on N_2_O Fluxes and productivity of lucerne grasslands on the Loess Plateau. (In Chinese, with English abstract.) Acta Agrestia Sinica, 2022,30(06):1584-1589.
68. Xu Ruizhi. Study on Effects of Topdressing after Cutting on Alfalfa in the Longdong Loess Plateau of Chinal. (In Chinese, with English abstract.) [Master’s thesis, Lanzhou University], 2022.
69. Lei Li, XuWeizhou, Jia Yuzhen, et al., Effects of N, P and K application on traits, yield and nutritional quality of alfalfa in sandy areas of Yulin. (In Chinese, with English abstract.) FEED RESEARCH 2021,44(19):116-120.
70. Ji Chao, Yin Guomei, Liu Sibo, et al., Effect of nitrogen levels on alfalfa agronomic traits and economic benefit. (In Chinese, with English abstract.) JOURNAL OF NORTHERN AGRICULTURE, 2021,49(05):127-134.
71. Meng Xuanchen, Ma Pengcheng, Ma Jie, et al., Study on the effect of mulching furrow and fertilization of alfalfa in semi-arid areasof loess plateau. (In Chinese, with English abstract.) Acta Agrestia Sinica, 2021,29(09):2098-2106.
72. Wu yong. Study on Fertilization responses of High Efficiency production of Alfalfa in the Irrigation Area of Hexi. (In Chinese, with English abstract.) [Master’s thesis, Gansu Agricultural University], 2021.
73. Li Ning. Effects of Nitrogen and Phosphorus Fertilizers on NutritionalYield and Quality of Different Alfalfa Varieties. (In Chinese, with English abstract.) [Master’s thesis, Northwest Agriculture & Forestry University], 2021.
74. Liu Lin. Effects of fertilization on alfalfa productivity and soil microbial nutrient utilization characteristics. (In Chinese, with English abstract.) [Master’s thesis, Ningxia University], 2021.
75. Na Jia, Huang Lihua, Yan Yimin, et al., Response of alfalfa yield and quality to mowing frequency and fertilization in saline-sodic land of thewestern Songnen Plain. (In Chinese, with English abstract.) Journal of Agricultural Resources and Environment, 2021,38(05):882-890.
76. Tong Changchun, Liu Xiaojing, Lin Fang, et al., Yield effect of optimisation of photosynthetic characteristics of alfalfa through balanced fertilization. (In Chinese, with English abstract.) Acta Prataculturae Sinica, 2020,29(08):70-80.
77. Ma Tiecheng, Zhang Hui. Effect of nitrogen and phosphorus fertilizer application on the growth index and yield of alfalfa in A Wei irrigation area. (In Chinese, with English abstract.) Xinjiang Agricultural Sciences ,2020,57(08):1535-1541.
78. Sun Hao, Zhang Yuxia, Liang Qingwei, et al., Effects of fertilization on yield and quality of alfalfa in Horqin sand land. (In Chinese, with English abstract.) Grassland and Turf, 2020,40(03):30-41.
79. Qiao Zimei. Effects Of Irrigation And Fertilization On The Productivity And Overwintering Of Alfalfa Grassland In The Alpine Desert Area Of China. (In Chinese, with English abstract.) [Master’s thesis, Lanzhou University], 2021.
80. Yang Mei. Study on leaf trait responses and mechanisms of phosphatases regulating leaf P resorption of lucerne under P and K fertilization with water supply. (In Chinese, with English abstract.) [Doctoral dissertation, Lanzhou University], 2021.
81. Chen Xianglai, Pan Jia, Chen Lijun, et al., Effects of fertilization on hay yield and quality of alfalfa on the Loess Plateau. (In Chinese, with English abstract.) Pratacultual Science, 2019,36(12):3145-3154.
82. He Fei, Zhao Zhongxiang, Kang Junmei, et al., Effects of N,P and K fertilizer on alfalfa hay yield and quality. (In Chinese, with English abstract.) Chinese Journal of Grassland, 2019,41(05):24-32.
83. Miao Xiaorong. Effects of nitrogen and phosphorus and microelement fertilizer on productivity of alfalfa under drip irrigation. (In Chinese, with English abstract.) [Master’s thesis, Shihezi University], 2019.
84. Hou Siyu.Effects of nitrogen and phosphorus application on bud bank, yield and quality in alfalfa. (In Chinese, with English abstract.) [Master’s thesis, Northeast Normal University], 2019.
85. Zhang Qian,Li Jinsheng,Zhao Tianci, et al., Effects of different fertilizer types and ratios on alfalfa productivity and quality in sandy soil. (In Chinese, with English abstract.) Acta Agrestia Sinica,,2019,27(02):383-388.
86. Xiao Yanzi,Wurenqiqige,Meng Kai,, et al., Effect of formula fertilizer on the productivity of alfalfa. (In Chinese, with English abstract.) Chinese Journal of Grassland, 2020,42(01):174-178.
